# Is endothelial dysfunction induced by aromatase inhibitors reversible after treatment?

**DOI:** 10.1007/s10549-026-08007-2

**Published:** 2026-06-22

**Authors:** Mohamed S. Dabour, Adnan Shaaban, Daniel A. Duprez, Beshay N. Zordoky, Anne H. Blaes

**Affiliations:** 1 Department of Experimental and Clinical Pharmacology, College of Pharmacy, University of Minnesota, Minneapolis, MN USA; 2 Department of Clinical Pharmacy, Faculty of Pharmacy, Tanta University, Tanta, Egypt; 3 Division of Cardiology, AdventHealth Orlando, Orlando, FL USA; 4 Division of Cardiology, University of Minnesota, Minneapolis, MN USA; 5 Division of Hematology/Oncology/Transplantation, Medical School, University of Minnesota, 909 Fulton St SE, Minneapolis, MN 55455 USA

**Keywords:** Breast cancer survivorship, Aromatase inhibitors, Endothelial, EndoPAT, Cardiovascular toxicity, Vascular function

## Abstract

**Purpose:**

Aromatase inhibitors (AIs) are standard therapy for postmenopausal women with hormone receptor-positive breast cancer. However, prolonged AI use is associated with increased cardiovascular (CV) risk, including hypertension, and endothelial dysfunction. This study evaluated the longitudinal changes in endothelial function during AI therapy, and whether AI-induced endothelial dysfunction is reversible post-discontinuation.

**Methods:**

Patients were recruited before or within one month of AI initiation (Pre/early AI), during AI therapy, and after discontinuation (Post-AI) from two prospective studies. Patients with hypertension, hyperlipidemia, diabetes, or tobacco use were excluded. Vascular assessments included peripheral arterial tonometry (EndoPAT) for endothelial function (abnormal if ratio < 1.67) and artery elasticity indices. Estradiol, lipid profiles, and inflammatory markers were also measured.

**Results:**

The study included 12 Pre/early AI patients, 67 visits from 41 patients during AI (median 2.89 years on AI), and 9 Post-AI patients (median 4.17 years follow-up). EndoPAT ratio was significantly impaired during AI therapy compared to the Pre/early AI (median: 0.86 vs 2.19). The EndoPAT ratio declined as early as six months and showed a progressive decline over time. Post-discontinuation, the EndoPAT ratio was only partially and not significantly restored (median: 1.08) despite full estradiol restoration and long follow-up. Arterial elasticity showed no significant changes. Systolic blood pressure increased modestly during AI therapy and returned to baseline after discontinuation, while diastolic pressure remained unchanged. Circulating interleukin-6 and tumor necrosis factor-α significantly decreased following AI discontinuation.

**Conclusions:**

AI therapy is associated with significant and progressive endothelial dysfunction, which does not fully recover after treatment cessation, highlighting the importance of CV monitoring in patients receiving long-term AI therapy.

**Supplementary Information:**

The online version contains supplementary material available at 10.1007/s10549-026-08007-2.

## Introduction

Incidence of breast cancer has consistently increased, now accounting for approximately 32% of all newly diagnosed cancers and ranking as the second leading cause of cancer-related mortality among women, following lung cancer [1]. Significant improvements in breast cancer survival rates have occurred in recent years due to earlier detection and advances in treatment, including the use of aromatase inhibitors (AIs). AIs have become a standard treatment for postmenopausal women with hormone receptor-positive breast cancer, as they have been shown to improve disease-free survival and overall survival, and to reduce the risk of recurrence compared to tamoxifen in postmenopausal women [2]. AIs work by inhibiting the enzyme aromatase, which is the final step in the biosynthesis of estrogen, thus depriving estrogen receptor-positive breast cancer cells of the estrogen necessary for their growth and proliferation. Five years of AI therapy is the standard initial duration for most postmenopausal women, with extended therapy considered for those at high risk of late recurrence [2]. However, extended use of AIs has been associated with increased the risk for cardiovascular adverse events [3]. Patients treated with aromatase inhibitors are more susceptible to develop hyperlipidemia, hypercholesterolemia and hypertension, all of which are established risk factors for cardiovascular disease, in comparison to those receiving tamoxifen [4]. This increased cardiovascular risk is attributed to the peripheral inhibition of estrogen by AIs, which deprives the endothelium of the protective effects of estrogen. Estrogen plays a critical role in maintaining vascular homeostasis by enhancing endothelium-dependent vasodilation by increasing nitric oxide production and bioavailability, reducing vascular inflammation [5], and improving lipoprotein metabolism [6], all contributing to cardiovascular protection. We previously demonstrated that treatment of postmenopausal breast cancer with AIs is associated with a reduction in endothelial function within the first six months of therapy, which coincides with declines in estrogen levels after initiating the medication [7]. The aim of the current study was to evaluate the longitudinal trajectory of endothelial function during prolonged AI therapy and, importantly, to determine whether AI-associated endothelial dysfunction is reversible following discontinuation of AIs.

## Methods

### Subjects

Patients in this report were included from two prospective studies: Aromatase Inhibitors and Vascular Health (AIVH) and Vascular Assessment in Breast Cancer Survivors Taking Aromatase Inhibitors (VABC). In the VABC study, patients completed two visits: one before or within one month of starting an AI (Pre/early AI), and a second visit six months to one year later while on AI. In the AIVH study, patients had visits at several time points during AI therapy. Nine patients also had a visit after stopping the aromatase inhibitor (post-AI, seven from AIVH and two from VABC). Together, these complementary studies allowed for the classification of participants into three categories: Pre/early AI, AI, and Post-AI. Eligible patients were adults (≥18 years) with estrogen- or progesterone-positive breast cancer, postmenopausal either naturally or medically, and prescribed an AI as part of treatment. Patients were excluded if they had a history of myocardial infarction, congestive heart failure, cardiac catheterization with intervention, hypercholesterolemia, were taking antihypertensive or cholesterol-lowering medications, or were current smokers. Both studies were approved by the University of Minnesota Institutional Review Board and Cancer Center Review Committee, and all patients provided written informed consent according to the Declaration of Helsinki.

### Vascular assessment

Vascular function was assessed using two non-invasive, standardized techniques: Pulse Wave Analysis (PWA) and Peripheral Arterial Tonometry (PAT) as previously described [8, 9]. PWA was conducted using the HDI/PulseWave CR-2000 Cardiovascular Profiling System, which employs an applanation tonometer to measure radial arterial pulse waveforms. These waveforms are subsequently digitized, analyzed, and stored electronically. Large artery elasticity (LAE) and small artery elasticity (SAE) indices are derived from the diastolic pulse contour analysis based on a modified Windkessel model. LAE and SAE were shown to be predictive of heart failure events, with SAE predictive of coronary heart disease events, independent of arterial blood pressure [10].

Endothelial function was assessed via PAT using the EndoPAT 2000 system, a non-invasive, FDA-approved device that measures digital vascular tone following reactive hyperemia. The procedure involved a 5-minute supra-systolic occlusion of the brachial artery, followed by measurement of pulse wave amplitude at the fingertip during reactive hyperemia. The hyperemic response was compared to pre-occlusion baseline values and normalized to the contralateral non-ischemic control arm to calculate the Reactive Hyperemia Index (RHI, or EndoPAT ratio). Pressure signals were stored and analyzed by an automated computer algorithm to minimize intra- and inter-observer variability. Notably, an EndoPAT ratio < 1.67 has been associated with increased risk of adverse cardiac events, independent of traditional cardiovascular risk factors [9].

All vascular function testing (PWA and PAT) was performed with the participant in a supine position, at rest, in a quiet, air-conditioned room. To ensure standardized conditions, participants were required to fast for a minimum of 8 to 12 hours prior to the study. All vasoactive medications were withheld for at least four half-lives, where clinically feasible. Furthermore, participants were instructed to abstain from exercise, caffeine, high-fat foods, Vitamin C supplements, and tobacco use for at least 4 to 6 hours before the test. All testing was completed by the same technician to reduce inter-technician variability. Vascular assessments were performed by a single trained vascular technician (NF). Each assessment was conducted in triplicate, and the mean value of the three readings was used for analysis.

### Biomarker assessment

Fasting blood samples were collected to assess a panel of lipid and inflammatory biomarkers, including serum ultrasensitive estradiol, total cholesterol (TC), low-density lipoprotein (LDL), high-density lipoprotein (HDL), very low-density lipoprotein (VLDL), triglycerides (TG), high-sensitivity C-reactive protein (hs-CRP), fibrinogen, interleukin-6 (IL-6), and tumor necrosis factor-alpha (TNF-α). Blood draws were performed prior to vascular function testing. All samples were processed and aliquoted according to standardized protocols for subsequent biomarker analysis.

Inflammatory biomarkers were analyzed at the University of Minnesota Cytokine Reference Laboratory using a combination of multiplex Luminex assays and enzyme-linked immunosorbent assays (ELISA). Fibrinogen and hs-CRP were measured using multiplex Luminex assays, while other biomarkers were quantified using individual ELISA-based methods. Fasting lipid profiles and serum ultrasensitive estradiol concentrations were measured using standard clinical laboratory assays at Fairview Diagnostic Laboratories.

### Statistical analysis

Demographic and clinical characteristics of participants were summarized by group. For measurements obtained in triplicate, the average was used for analysis. Comparisons between pre/early AI, AI, and post-AI groups were performed using generalized estimating equations (GEE) with an independence working correlation structure and robust (sandwich) variance estimators to account for repeated measurements within participants because multiple measurements were obtained from the same participant over time. Each outcome, including EndoPAT ratio, systolic blood pressure (SBP), diastolic blood pressure (DBP), LAE, SAE, estradiol, IL-6 and TNF-α was modeled separately. Pairwise comparisons between groups were derived from the fitted models, and two-sided *p*-values were reported. *p*-values were visualized using GraphPad Prism (version 10.2.3, San Diego, CA, USA) for clarity. Descriptive data are presented as medians with interquartile ranges (IQR), while model-based estimates derived from GEE analyses are presented as estimated means with 95% confidence intervals (CI). GEE modeling was performed using R (version 4.3.1, R Foundation for Statistical Computing, Vienna, Austria). To evaluate the longitudinal effect of AI exposure duration on endothelial function, a secondary GEE model was constructed for EndoPAT that incorporated both AI exposure status and time on AI (in years), with time modeled as a continuous variable limited to On-AI observations. Pre/early AI and Post-AI observations were included without a time component. Model-based predictions of EndoPAT values were generated for clinically relevant time points (Pre/early AI, 6 months on AI, 2 years on AI, 5 years on AI, and Post-AI) and group differences were estimated using linear contrasts, with corresponding 95% CI and *p*-values.

## Results

### Patient characteristics

Patient characteristics at pre/early AI, during AI, and post-AI therapy are summarized in Table 1. A total of 12 patients were included in the pre/early AI analysis, with measurements during AI collected from 67 visits including 41 patients, and post-AI data available for 9 patients. The median time on AI in the pre/early AI group was 0.00 years (IQR: −0.04 to 0.03), consistent with minimal or no exposure at baseline. During AI therapy, the median time on treatment was 2.89 years (IQR: 0.71–5.52), while the median time since AI discontinuation in the post-AI group was 4.17 years (IQR: 3.35–4.72). The median age of patients at the pre/early AI visit was 58 years (interquartile range [IQR]: 56–60), which increased to 60 years (IQR: 58–66) during AI treatment and 67 years (IQR: 64–67) post-AI. Median body mass index (BMI) decreased over time, from 26.4 kg/m^2^ (IQR: 24.2–31.6) at pre/early AI to 24.25 kg/m^2^ (IQR: 21.68–30.73) during AI therapy and 22.4 kg/m^2^ (IQR 19.4–26.8) at the post-AI visit. Inflammatory markers, hs-CRP and fibrinogen, showed variability across visits. Median hs-CRP levels decreased from 2,451 ng/mL (IQR: 836–6,007) pre/early AI to 1,492 ng/mL (IQR: 839–3,177) during AI treatment, and were 1,613 ng/mL (IQR: 873.1–4,880) post-AI. Fibrinogen levels also decreased from 3,394 µg/ml (IQR: 2,811–3,666) pre/early AI to 2,835 µg/ml (IQR: 2,355–3,240) during AI, though post-AI data for fibrinogen was not available. Lipid parameters showed modest changes across visits. Median total cholesterol decreased from 227.5 mg/dL (IQR: 200–241.8) at pre/early AI to 219 mg/dL (IQR: 204–244) during AI therapy and 197.5 mg/dL (IQR: 185.5–235.5) at post-AI, with similar declines observed for LDL (142 mg/dL [117.3–163.3] pre/early AI; 126 mg/dL [111–147] during AI therapy; 121 mg/dL [99–153.5] post-AI) and triglycerides (90.5 mg/dL [72.25–125.5] pre/early AI; 77 mg/dL [59–102] during AI therapy; 69.5 mg/dL [49.75–81.5] post-AI). HDL remained relatively stable across visits, and the total cholesterol-to-HDL ratio showed minimal variation.

**Table 1:** Patient characteristics at pre/early AI visit, AI visits, and post-AI visit

Characteristic	Pre/early AI	N	AI	N	Post AI	N
Study		12		67		9
AIVH	0		25		7	
VABC	12		16		2	
Time on AI/post-AI (years)	0.00 (−0.04–0.03)	12	2.89 (0.71–5.52)	53	4.17 (3.35–4.72)^†^	9
Age (years)	58 (56–60)	12	60 (58–66)	67	67 (64–67)	9
BMI (kg/m^2^)	26.4 (24.2–31.6)	11	24.25 (21.68–30.73)	42	22.4 (19.4–26.8)	7
hs-CRP (ng/mL)	2,451 (836–6,007)	11	1492 (839–3177)	56	1,613 (873.1–4,880)	9
Fibrinogen (µg/ml)	3,394 (2,811–3,666)	11	2,835 (2,355–3240)	56	NA (NA – NA)	0
Total Cholesterol (mg/dL)	227.5 (200–241.8)	12	219 (204–244)	65	197.5 (185.5–235.5)	8
Triglycerides (mg/dL)	90.5 (72.25–125.5)	12	77 (59–102)	65	69.5 (49.75–81.5)	8
HDL (mg/dL)	63.5 (48.75–71.25)	12	71 (63–81)	65	67 (55.25–89.75)	8
LDL (mg/dL)	142 (117.3–163.3)	12	126 (111–147)	65	121 (99–153.5)	8
VLHDL	17 (14–24)	9	15 (13–21)	27	NA (NA – NA)	0
Cholesterol/HDL Ratio	3.85 (2.95–4.50)	12	3.00 (2.60–3.30)	62	3 (2.7–3.4)	8
Non-HDL Cholesterol (mg/dL)	202 (166–204)	3	157 (138–193)	15	134.5 (113–171)	8

Values are presented as median (interquartile range). N indicates the number of participants with available data for each characteristic. AI measurements included 67 visits from 41 patients. NA indicates data not available. ^†^ The median (IQR) time since AI discontinuation in the post-AI group

Breast cancer stage, chemotherapy exposure, and AI exposure are presented in Table 2. The majority of patients had stage I breast cancer, with 8 patients (50.0%) in the VABC group and 13 patients (59.1%) in the AIVH group. This was followed by stage II breast cancer, in 7 patients (43.8%) in the VABC group and 6 patients (27.3%) in the AIVH group. Fewer patients presented with stage III breast cancer. A total of 14 patients had received prior chemotherapy, including 5 patients (31.3%) in the VABC cohort and 9 patients (36.0%) in the AIVH cohort. Letrozole and anastrozole were the most frequently prescribed AIs in both cohorts. In the VABC cohort, 43.8% of patients received letrozole and 31.3% received anastrozole, whereas in the AIVH cohort, 40.0% received letrozole and 36.0% received anastrozole. Exemestane was less commonly prescribed. A subset of patients switched between different AIs during AI therapy, including anastrozole, letrozole, and exemestane, comprising 4 patients (25.0%) in the VABC group and 4 patients (16.0%) in the AIVH group. No patients in either cohort were prescribed CDK 4,6 inhibitors.

**Table 2:** Breast cancer stage and aromatase inhibitor exposure

Characteristic	VABC (n = 16)	AIVH (n = 25)
**Breast cancer stage**		
Stage I	8 (50.0)	13 (59.1)
Stage II	7 (43.8)	6 (27.3)
Stage III	1 (6.3)	3 (13.6)
**Chemotherapy exposure**		
Yes	5 (31.3)	9 (36.0)
No	11 (68.7)	16 (64.0)
**Aromatase inhibitor**		
Anastrozole	5 (31.3)	9 (36.0)
Letrozole	7 (43.8)	10 (40.0)
Exemestane	0 (0.0)	2 (8.0)
Switched between AIs	4 (25.0)	4 (16.0)

Values are n (%)

### Vascular assessment

**Fig. 1:**
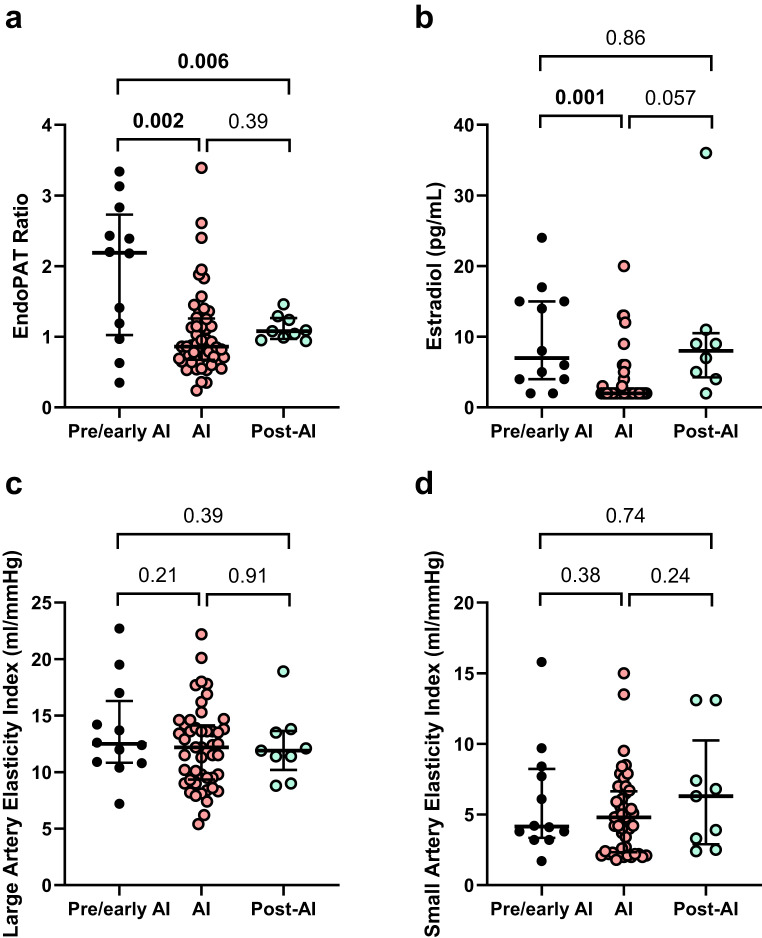
Vascular assessment and estradiol levels across aromatase inhibitor (AI) treatment phases. (**a**) Endothelial function measured by EndoPAT ratio (**b**) Serum estradiol concentrations, (**c**) Large artery elasticity index (LAE), and (**d**) Small artery elasticity index (SAE), assessed before AI treatment or within one month of starting an AI (Pre/early AI), during AI therapy (AI), and after AI (post-AI). Data are presented as median with IQR. Statistical analyses were performed using generalized estimating equations with an independence working correlation matrix and robust standard errors. *p*-values marked in bold indicate statistical significance

Endothelial function assessed by EndoPAT ratio, was significantly impaired during AI therapy compared to the Pre/early AI (Pre/early AI median: 2.19; AI median: 0.86; *p* = 0.002, Fig. 1a). Importantly, after AI discontinuation, the EndoPAT ratio was only partially and not significantly restored (median: 1.08) despite the full restoration of estradiol levels (Fig. 1b) and the long post-treatment follow-up period (median: 4.17 years; range: 2.98–5.24). Estradiol levels were significantly reduced during AI therapy compared with pre/early AI levels (Fig. 1b), consistent with effective estrogen suppression. After AI discontinuation, estradiol concentrations increased relative to AI levels, approaching pre/early AI values. No significant differences were observed in LAE (Fig. 1c) and SAE (Fig. 1d) between groups.

Paired EndoPAT analyses were performed in participants with repeated measurements across study phases (Supplementary Fig. 1). These analyses included participants with paired measurements between Pre/early AI and AI phases (Supplementary Fig. 1a), AI and Post-AI phases (Supplementary Fig. 1b), and the subset of participants with measurements available across all three phases (Supplementary Fig. 1c). Although only two participants had measurements spanning all three phases, paired analyses demonstrated values and trends consistent with the primary cohort analysis, including reduced EndoPAT ratios during AI therapy and incomplete recovery to baseline levels following AI discontinuation.

**Fig. 2:**
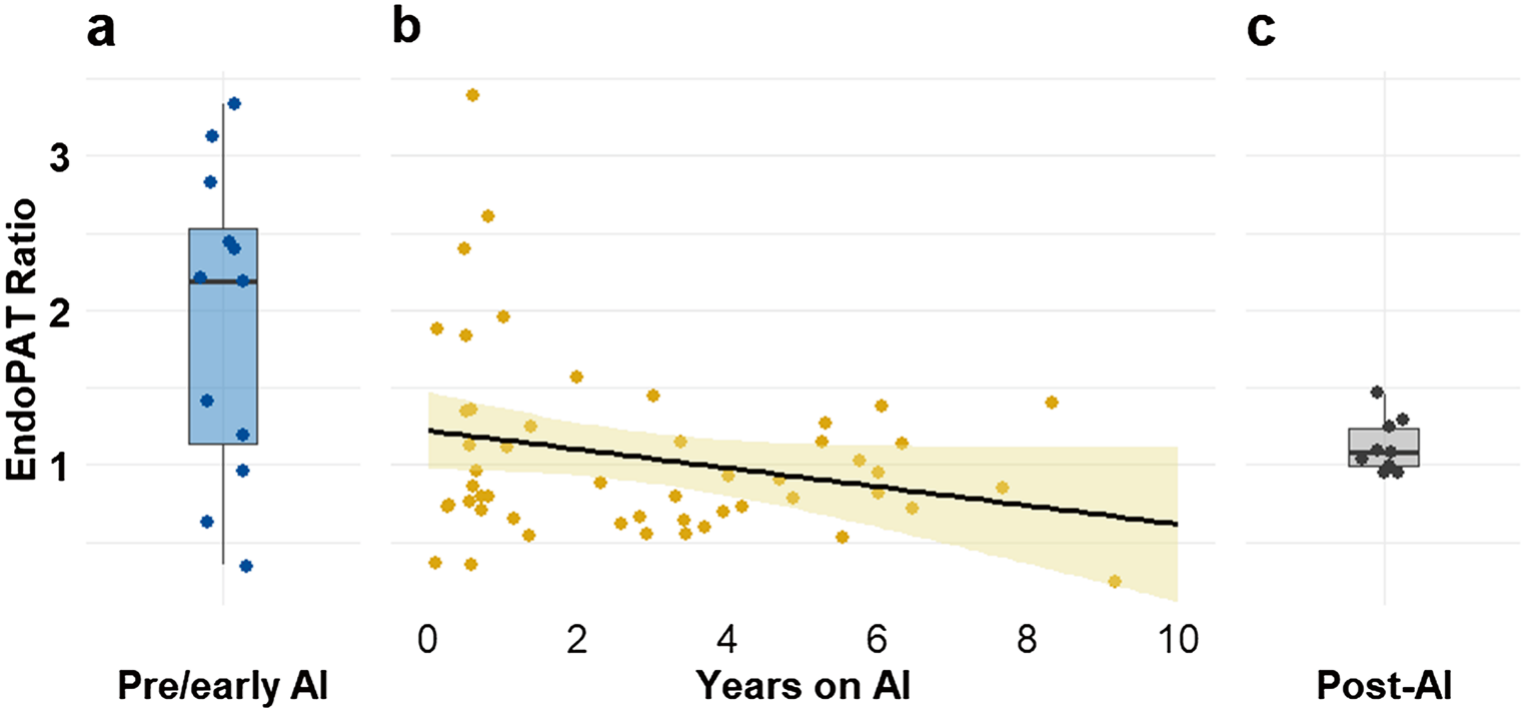
Longitudinal analysis of EndoPAT measurements by aromatase inhibitor (AI) treatment phase. Individual EndoPAT ratio measurements are shown. In panels (**a**) and (**c**), boxplots represent the median and interquartile range of observed values. In panel (**b**), the solid line represents the model-based estimated longitudinal trend during AI therapy, with shaded areas indicating the 95% confidence interval. (**a**) Subjects prior to AI therapy or within one month of starting an AI (Pre/early AI). (**b**) Subjects on AI therapy, plotted as a function of time (years) since initiating their first AI treatment. (**c**) Subjects following AI discontinuation (Post-AI)

To further evaluate the longitudinal effect of AI exposure duration on endothelial function, a GEE-based predictive model incorporating time on AI therapy was constructed. Model-predicted EndoPAT values demonstrated a progressive decline in endothelial function with increasing duration of AI exposure (Fig. 2, Table 3). The EndoPAT ratio declined as early as 6 months on AI (model-based mean at 6 months: 1.19; *p* = 0.010) and showed a progressive decline with increasing duration of AI use, with further reductions observed at 2 years (mean: 1.10; *p* = 0.003) and 5 years on AI therapy (model-based mean: 0.92; mean difference: −1.00; *p* < 0.001).

**Table 3:** Estimated EndoPAT means and confidence intervals across AI treatment phases

					Comparison to Pre/early AI		
Status		Observed Median (IQR)	GEE-estimated Mean EndoPAT	95% CI	Mean Difference	95% CI	p-value
Pre/early AI		2.19 (1.14–2.53)	1.92	(1.39, 2.46)	-	-	-
6-months on AI			1.19	(0.90, 1.49)	−0.73	(−1.28, −0.18)	0.010
2 Years on AI			1.1	(0.89, 1.32)	−0.82	(−1.35, −0.29)	0.003
5 Years on AI			0.92	(0.79, 1.05)	−1	(−1.54, −0.46)	<0.001
Post-AI		1.08 (0.99–1.24)	1.12	(1.01, 1.23)	−0.8	(−1.35, −0.25)	0.004

Observed median EndoPAT values with interquartile ranges (IQR) are shown for the Pre/early AI and Post-AI groups. Estimates of the conditional mean and 95% confidence interval (CI) for EndoPAT ratio among subjects before AI treatment or within one month of starting an AI (Pre/early AI), at 6 months, 2 years, and 5 years on AI therapy, and after AI discontinuation (Post-AI). Conditional means were compared to the Pre/early AI group. Reported 95% CIs and *p*-values are unadjusted for multiple comparisons.

### Blood pressure measurements

**Fig. 3:**
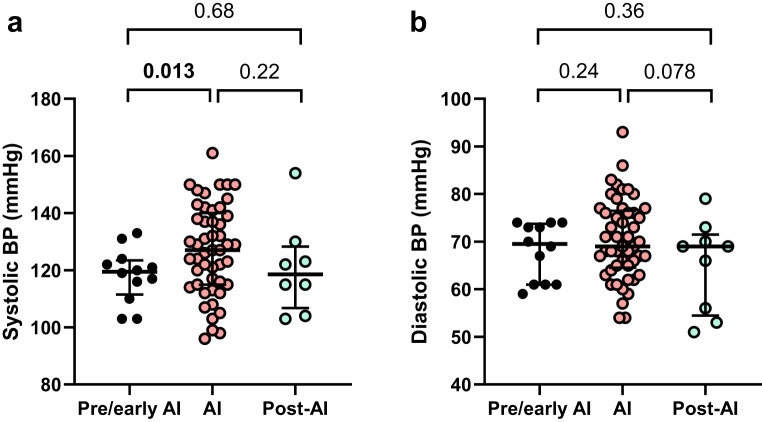
Blood pressure measurements across aromatase inhibitor (AI) treatment phases. (**a**) Systolic blood pressure (SBP) and (**b**) Diastolic blood pressure (DBP) measured before AI treatment or within one month of starting an AI (Pre/early AI), during AI therapy (AI), and after AI discontinuation (Post-AI). Data are presented as median with IQR. Statistical analyses were performed using generalized estimating equations with an independence working correlation matrix and robust standard errors. *p*-values marked in bold indicate statistical significance

SBP increased significantly but modestly from 119.5 (IQR: 111.5–123.5) mmHg in the Pre/early AI group to 127.0 (IQR: 115.0–140.0) mmHg during AI therapy (Fig. 3a). Following AI discontinuation, SBP decreased to 120.8 ± 16.3 mmHg, which was not significantly different from either the AI group or the Pre/early AI group. DBP did not significantly differ between groups (Fig. 3b).

### Inflammatory markers assessment

**Fig. 4:**
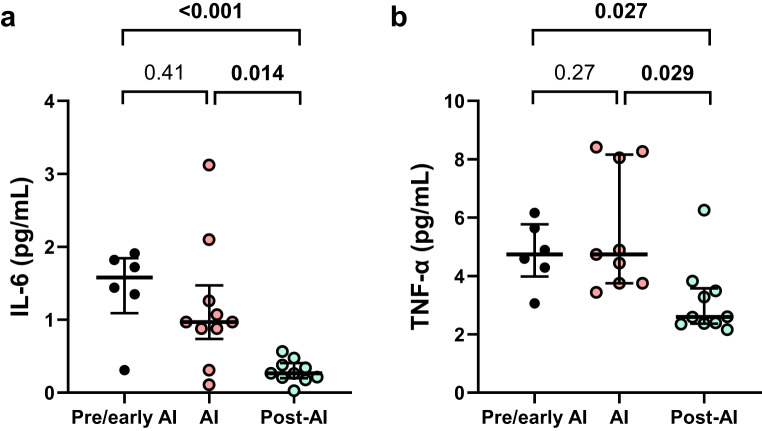
Inflammatory markers assessment across aromatase inhibitor (AI) treatment phases. (**a**) Interleukin-6 (IL-6) and (**b**) tumor necrosis factor-α (TNF-α) measured before AI treatment or within one month of starting an AI (Pre/early AI), during AI therapy (AI), and after AI discontinuation (Post-AI). Data are presented as median with IQR. Statistical analyses were performed using generalized estimating equations with an independence working correlation matrix and robust standard errors. *p*-values marked in bold indicate statistical significance

Circulating levels of the inflammatory cytokines IL-6 and TNF-α were assessed. Both IL-6 (Fig. 4a), and TNF-α (Fig. 4b) were elevated during the Pre/early AI phase and remained elevated during AI treatment, while significantly declined following treatment completion in the post-AI phase. No significant difference was observed between the Pre/early AI and AI phases.

## Discussion

The population of breast cancer survivors is expanding, yet they face a significant burden of comorbidities [11, 12]. Approximately 80% of breast cancer cases are hormone receptor-positive, for which endocrine therapy is a primary treatment modality [13]. While AIs and tamoxifen represent the two principal endocrine therapies, AIs are generally associated with favorable clinical outcomes [14]. However, the risk of AI-related cardiovascular disease has emerged as a growing concern. Extensive clinical evidence indicates a higher prevalence of hypertension, heart failure, hypercholesterolemia, and ischemic cardiovascular events among postmenopausal survivors receiving AI treatment [3, 4]. Recent population-based studies have reported a significantly increased risk of heart failure and cardiovascular mortality in patients treated with AIs compared to those receiving tamoxifen, with nonsignificant trends toward higher rates of myocardial infarction and ischemic stroke [15]. A recent systematic review and meta-analysis confirmed a higher risk of overall cardiovascular disease for patients on AI therapy relative to those receiving tamoxifen [16]. This is particularly concerning given that breast cancer survivors already face an increased risk of cardiovascular disease-related mortality compared to women without breast cancer, a risk that becomes especially evident approximately seven years after diagnosis [17].

Cardiovascular disease develops through a multifactorial process involving genetic predisposition, aging, lifestyle factors, and common comorbidities such as hypertension, dyslipidemia, and diabetes [18]. A critical early step in this progression is endothelial dysfunction, which compromises vascular homeostasis and sets the stage for atherosclerosis and subsequent ischemic events [18]. In postmenopausal women, estrogen deficiency further exacerbates endothelial injury due to the loss of the protective vascular effects of estrogen [5]. Endothelial dysfunction is an independent predictor of cardiovascular events, independent of traditional cardiac risk factors [19]. Notably, growing evidence supports the prognostic value of endothelial function in predicting cardiovascular risk. For example, an EndoPAT index below 1.67 is associated with the adverse cardiovascular outcomes [20]. Moreover, individuals with reduced EndoPAT indices have been shown to experience higher rates of adverse cardiac events over long-term follow-up, even in the absence of overt symptoms [21].

In the current study of postmenopausal breast cancer survivors on AI therapy, we observed a significant decline in endothelial function during AI treatment, as measured by the EndoPAT reactive hyperemia index. EndoPAT ratios decreased from a mean of 1.9 at baseline (pre/early AI) to 1.03 during AI therapy, consistent with endothelial dysfunction. Notably, this impairment persisted even after AI discontinuation, where the EndoPAT ratio remained low (1.12) and was not significantly restored compared to baseline, despite full recovery of serum estradiol levels post-AI. Furthermore, our longitudinal time-on-AI analysis demonstrated that endothelial function worsened progressively with longer AI duration, where a significant decline was evident by 6 months, with further reductions at 2 years and 5 years of AI use. In contrast, we found no significant longitudinal changes in LAE or SAE during AI treatment.

Our findings build upon emerging evidence that AI therapy impair endothelial function. Blaes et al. first reported in a cross-sectional pilot study that postmenopausal women on AIs had significantly lower endothelial function than controls (EndoPAT ratio median 0.8 vs 2.7; *p* < 0.001) [9]. The study also noted trends toward reduced arterial elasticity in AI-treated women, though not statistically significant, which aligns with our results of no significant change in LAE/SAE despite obvious endothelial impairment. A subsequent short-term prospective study observed that over 6–12 months, a significantly greater proportion of women on AIs experienced a ≥20% deterioration in EndoPAT ratio compared to women not on Ais [22]. Consistent with these findings, we previously demonstrated an acute reduction in endothelial function during the first 6 months of AI therapy [7]. The current study extends these observations by capturing the longitudinal trajectory of vascular function over multiple years of AI use and after discontinuation, reflecting the real-world administration of Ais in those with breast cancer. We showed a time-dependent cumulative effect where endothelial function declined early and continued to worsen with prolonged AI duration. We have now shown that even after a median of 4 years since AI cessation, endothelial function remained substantially below pre-treatment levels, indicating only a partial endothelial recovery.

AIs act by suppressing aromatase activity and estradiol production, thereby depriving the endothelium of the protective effects of estrogen, which is known to promote endothelium-dependent vasodilation through the enhancement of nitric oxide (NO) production, reduced oxidative stress, and modulation of inflammatory pathways [5]. The loss of these vasoprotective mechanisms during AI therapy provides a plausible explanation for the decline in endothelial function. Prolonged estrogen deprivation may result in the loss of ERα-mediated activation of endothelial NO synthase (eNOS), or may trigger accelerated vascular aging and remodeling that persists even after estrogen levels normalize [23, 24], potentially explaining why endothelial function did not return to baseline following AI discontinuation.

In this study, SBP increased significantly from 119.5 mmHg (pre/early AI) to 127.0 mmHg during AI therapy, while DBP remained unchanged. Notably, after discontinuing the AI, SBP declined to 121 mmHg. AIs have been associated with higher rates of hypertension in clinical trials [4] and cross-sectional data show women on AIs have higher mean SBP (128 mmHg) than controls (116 mmHg, *p* < 0.01) with no difference in diastolic pressure [9]. Estrogen normally has vasodilatory effects, so its reduction during AI therapy can impair endothelial function and increase arterial tone. Preclinical findings also support a causal link between estrogen deprivation and increased blood pressure for example, chronic anastrozole treatment in female rats increases blood pressure [25]. Since AIs are frequently prescribed for 5–10 years, even temporary elevations in blood pressure over such extended durations may contribute to long-term cardiovascular burden, particularly in postmenopausal women and breast cancer survivors who often have increased baseline cardiovascular risk. Therefore, regular monitoring is warranted, and clinicians should consider standard hypertension management strategies during AI therapy.

Our findings demonstrated that IL-6 and TNF-α were elevated before adjuvant AI therapy, remained persistently high during active AI treatment, and then declined significantly after completing therapy. While prior evidence that IL-6 and TNF-α levels are often elevated in women with active breast cancer [26], the current study primarily included patients with early-stage disease. Therefore, the observed inflammatory profile may reflect residual systemic effects of prior active malignancy, host-related factors, or other underlying processes rather than active tumor burden. Importantly, the lack of further rise in IL-6 or TNF-α during AI therapy implies that estrogen deprivation per se did not exacerbate systemic inflammation.

Participants in the post-AI group had lower BMI compared with earlier time points, which may have contributed to the observed reductions in blood pressure and inflammatory biomarkers. Weight loss has been consistently associated with improvements in cardiovascular risk factors, including reductions in systolic blood pressure and circulating inflammatory markers [27]. However, previous studies suggest that weight loss generally results in either no statistically significant change or only modest improvement in endothelial function [28, 29]. Therefore, the persistence of impaired EndoPAT despite lower BMI suggests that endothelial dysfunction is unlikely to be explained by weight change and may instead reflect sustained effects of estrogen deprivation. Importantly, the lower BMI observed at later time points would be expected to bias toward improved, rather than worsened, endothelial function.

Because of the longitudinal study design, participants in the post-AI group were generally older than those in the Pre/early AI and AI groups. Aging has been associated with gradual age-related changes in endothelial function, including modest reductions in EndoPAT ratio. In a cohort of community-dwelling elderly women with a mean age of approximately 75.3 years, which is older than our cohort (median age: 67 years), EndoPAT values ranged from 1.54 to 1.77, values that are near or above the threshold of 1.67 considered indicative of normal endothelial function [30]. In contrast, although endothelial function in our post-AI group demonstrated partial and incomplete recovery over time, EndoPAT ratios remained abnormal and below the 1.67 threshold, with median values around 1.0–1.1 despite prolonged follow-up after AI discontinuation. These findings suggest that the magnitude of endothelial dysfunction observed in our cohort exceeds what is typically reported with aging alone, supporting the possibility that estrogen deprivation associated with AI therapy is a major contributor to endothelial dysfunction.

Several limitations of our study should be acknowledged. First, the sample size was relatively small, particularly for participants who completed post-AI follow-up. Second, the cohort was included from two separately designed studies, one that included a pre/early AI visit and another that did not. The Pre/Early AI group exhibited greater variability in EndoPAT measurements, likely due to the small sample size. Another limitation of this study is that not all participants contributed measurements across all study phases (Pre/early AI, AI, and Post-AI), limiting fully paired longitudinal comparisons within the same individuals. In addition, the prolonged duration of the longitudinal follow-up made complete follow-up across all study phases challenging. Although supplementary paired EndoPAT analyses demonstrated median values and trends consistent with the primary cohort findings, including reduced endothelial function during AI therapy and incomplete recovery following AI discontinuation, the number of participants with fully paired measurements across all three phases was limited. Future prospective longitudinal studies with fully paired repeated measurements across all treatment phases will be important to better define the temporal relationship between AI exposure and endothelial dysfunction within the same individuals.

In conclusion, this study demonstrates that AI-induced endothelial dysfunction is a progressive process that may not be fully reversible upon treatment cessation. The cardiovascular risks associated with AI therapy may persist into the survivorship period, independent of hormonal recovery. Future research should focus on identifying the mechanisms underlying this persistent dysfunction and evaluating interventions to mitigate long-term vascular damage in breast cancer survivors.

## Electronic supplementary material

Below is the link to the electronic supplementary material.

Supplementary Material 1

## Data Availability

Data available on reasonable request to the corresponding author.
