# Supplementary material for: Is endothelial dysfunction induced by aromatase inhibitors reversible after treatment?
Source: Breast Cancer Res Treat. 2026 Jun 22;217(3):55. doi: 10.1007/s10549-026-08007-2 (PMC13287299; doi:10.1007/s10549-026-08007-2)
Supplement: Supplementary file 1 — Supplementary Material 1 [file 10549_2026_8007_MOESM1_ESM.docx]

**Supplementary Fig. 1.** Paired longitudinal EndoPAT measurements across aromatase inhibitor (AI) treatment phases. (a) Participants with paired Pre/early AI (median: 2.19) and AI measurements (median: 1.05) (*n* =12). (b) Participants with paired AI (median: 0.72) and Post-AI measurements (median:1.06) (*n* =8). (c) Participants with measurements available across all three phases (Pre/early AI (median:2.76, AI (median: 0.91), and Post-AI (median: 1.085)) (*n* =2). Data are presented as median with IQR. Statistical comparisons were performed using paired t-tests.
